# Recovery of strength after reduced pediatric fractures of the forearm, wrist or hand; A prospective study

**DOI:** 10.1371/journal.pone.0230862

**Published:** 2020-04-01

**Authors:** Ann M. Hepping, Britt Barvelink, Joris J. W. Ploegmakers, Job van der Palen, Jan H. B. Geertzen, Sjoerd K. Bulstra, Jorrit S. Harbers, Martin Stevens

**Affiliations:** 1 Department of Rehabilitation Medicine, University Medical Center Groningen, University of Groningen, Groningen, the Netherlands; 2 Roessingh Center for Rehabilitation, Enschede, The Netherlands; 3 Department of Orthopedics, University Medical Center Groningen, University of Groningen, Groningen, the Netherlands; 4 Department of Research Methodology, Measurement and Data Analysis, University of Twente, Enschede, The Netherlands; 5 Medisch Spectrum Twente, Medical School Twente, Enschede, The Netherlands; 6 Department of Surgery, University Medical Center Groningen, University of Groningen, Groningen, The Netherlands; John Hunter Hospital and University of Newcastle, AUSTRALIA

## Abstract

**Introduction:**

The way strength recovers after reduction of pediatric fractures of the upper extremity has not previously been the specific scope of research. This is remarkable, since strength measurements are often used as an outcome measure in studies on trauma of the upper extremity. The aim of this study was to evaluate how strength recovers after sustainment of fractures of the forearm, wrist or hand treated by closed or open reduction in children and adolescents in the first 6 months after trauma. How much strength is lost at 6 weeks, 3 months and 6 months after trauma, and is this loss significant? Are there differences in the pattern of recovery between children who underwent a different treatment? And finally, which of the following factors are associated with an increase in the ratio between affected grip strength and expected strength: type of fracture, cast immobilization, occurrence of complications, and degree of pain?

**Design:**

Prospective observational study.

**Participants:**

Children and adolescents aged 4–18 years with a reduced fracture of the forearm, wrist or hand.

**Methods:**

Grip strength, key grip and three-jaw chuck grip were measured twice in each hand 6 weeks, 3 months and 6 months after trauma. Details on fracture type and location, treatment received, cast immobilization and complications were obtained. Hand-dominance and pain were verbally confirmed.

**Results:**

Loss of strength was more prominent and prolonged the more invasive the treatment, hence most extensive in the group receiving open reduction with internal fixation (ORIF), intermediate in the group receiving closed reduction with percutaneous pinning (CRIF), and least extensive in the group undergoing closed reduction without internal fixation (CR). Besides time passed, gender and age were of significant influence on strength, although there was no difference in pattern of recovery over time between children who received a different treatment. In the period of 6 weeks to 3 months after trauma, female gender, type of fracture sustained and occurrence of an unwanted event were associated with an increased ratio between affected and expected grip strength. For the later phase of recovery, between 3 and 6 months, this was only true for the occurrence of an unwanted event.

## Introduction

Within the extensive arsenal of existing functional tests, strength measurements are conducted almost routinely in the follow-up after trauma of the upper extremity in adults because of their well-established role in the assessment of hand function. Strength measurements are quick to assess and have excellent intra- and interrater reliability.[1–3]Scores of the affected hand are usually compared to those of the unaffected hand, or when available to reference values, in order to monitor disease activity, recovery and/or treatment efficacy.

Illustrative for the importance of strength measurements in the recovery of pediatric forearm fractures is the prospective study by Pershad et al. 2000.[4] Results showed a decrease in grip strength of 20% or more compared to the unaffected hand to be predictive for the presence of a fracture. The difference in grip strength between the fractured and the non-fractured group was found to be significant, whereas surprisingly the same did not hold true for range of motion of the wrist. However, within the field of pediatric traumatology or orthopedic surgery, strength measurements seem to be predominantly used as outcome parameters to compare two different types of treatment and/or in the setting of a long-term follow-up evaluation.[5–9]] Studies measuring strength shortly after trauma are extremely scarce.[5–8,10]Furthermore, we could not identify any studies that assessed recovery of strength itself in children after sustainment of reduced fractures. Comparing the affected hands between different treatment groups in itself gives no actual information about recovery of the individual children, as strength could very well still be diminished in the highest scoring group. More insight is needed into the recovery of strength in the first period after trauma, in particular in comparison to the unaffected hand.

The aim of this prospective study is to evaluate how strength recovers in children and adolescents after having sustained fractures of the forearm, wrist or hand treated by closed or open reduction. The research questions were as follows. How much strength is lost at 6 weeks, 3 months and 6 months after trauma, and is this difference significant in comparison to the unaffected hand? Are there differences in pattern of strength recovery between children treated by means of closed reduction (CR), closed reduction with percutaneous pinning (CRIF), and open reduction using either percutaneous pinning, intramedullary pinning or plate fixation (ORIF)? And finally, which of the following factors are associated with an increase in the ratio between affected grip strength and expected strength: type of fracture, cast immobilization, occurrence of complications, and degree of pain?

## Methods

### Study design

#### A prospective observational study

Children and their parents were informed about the study by one of the researchers (AMH/BB) and received additional written information about the study goals and procedures. Written consent was obtained from parents or the legal guardian. Children were only included if they themselves were willing to participate, and the researcher made sure parents as well as children knew that participation was neither mandatory nor would affect their treatment. The study received a waiver from the Medical Ethical Board of University Medical Center Groningen (M.14.150324).

### Participants and procedures

All children and adolescents aged 4–18 years with a reduced fracture distal from the olecranon treated at University Medical Center Groningen in a one year period were invited to participate. Exclusion criteria comprised neuromuscular and bone diseases, any condition interfering with normal growth, and fractures proven or suspected to be the result of child abuse. Also excluded were children who could not be properly instructed, for example due to a language barrier, or who received follow-up at a different hospital. Participating children had 3 appointments: at 6 weeks (T1), 3 months (T2) and 6 months (T3) after sustainment of the fracture. Participants were not measured in the week following cast or osteosynthesis removal. In those cases measurements were postponed. When appointments at the hospital could not be planned concurrently with measurement sessions, a home visit by the researcher was offered. Patients were assigned to each treatment regimen by the treating physician as part of the standard-of-care.

### Outcome measurements

**Fig 1:**
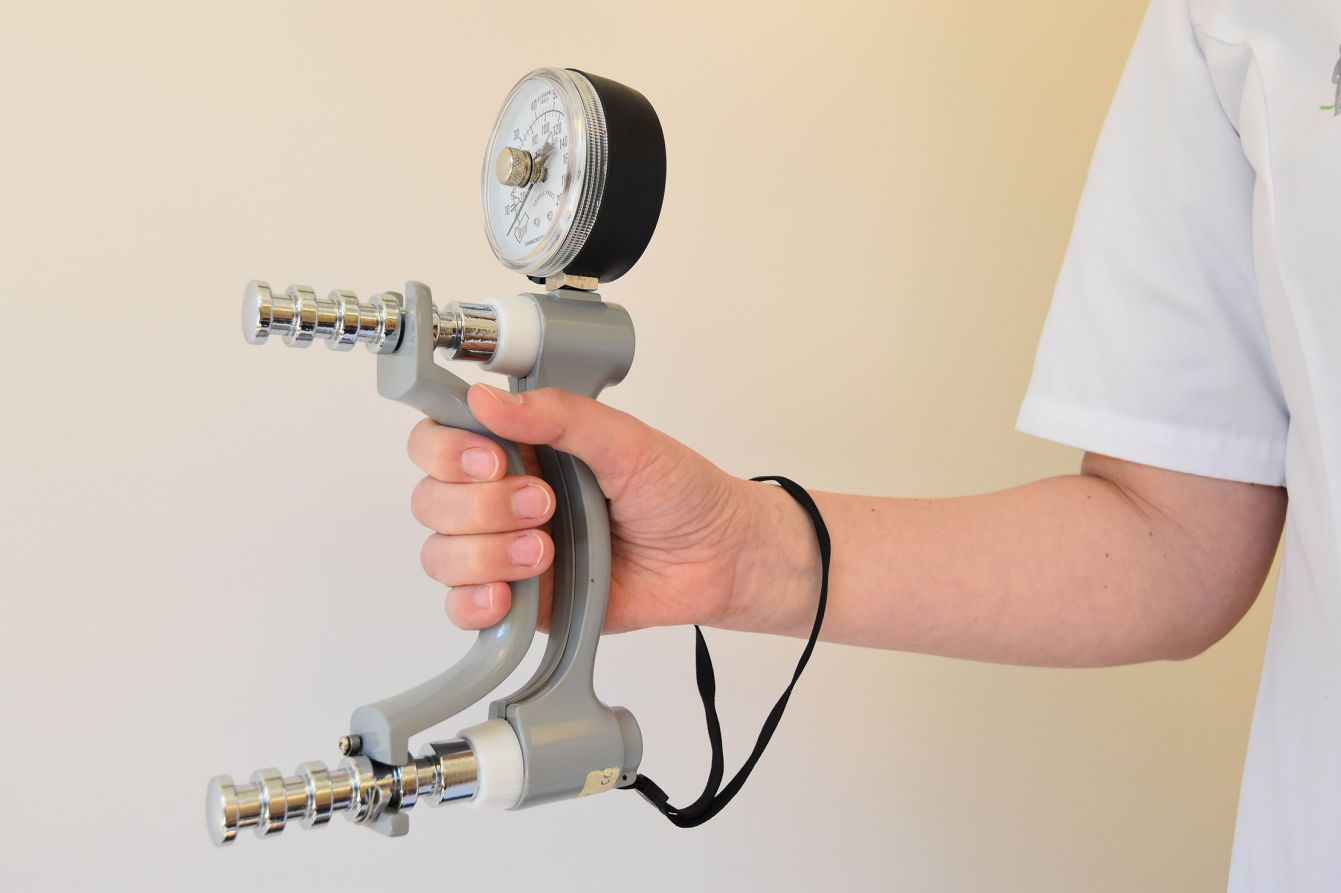
Photo of grip strength grasp.

**Fig 2:**
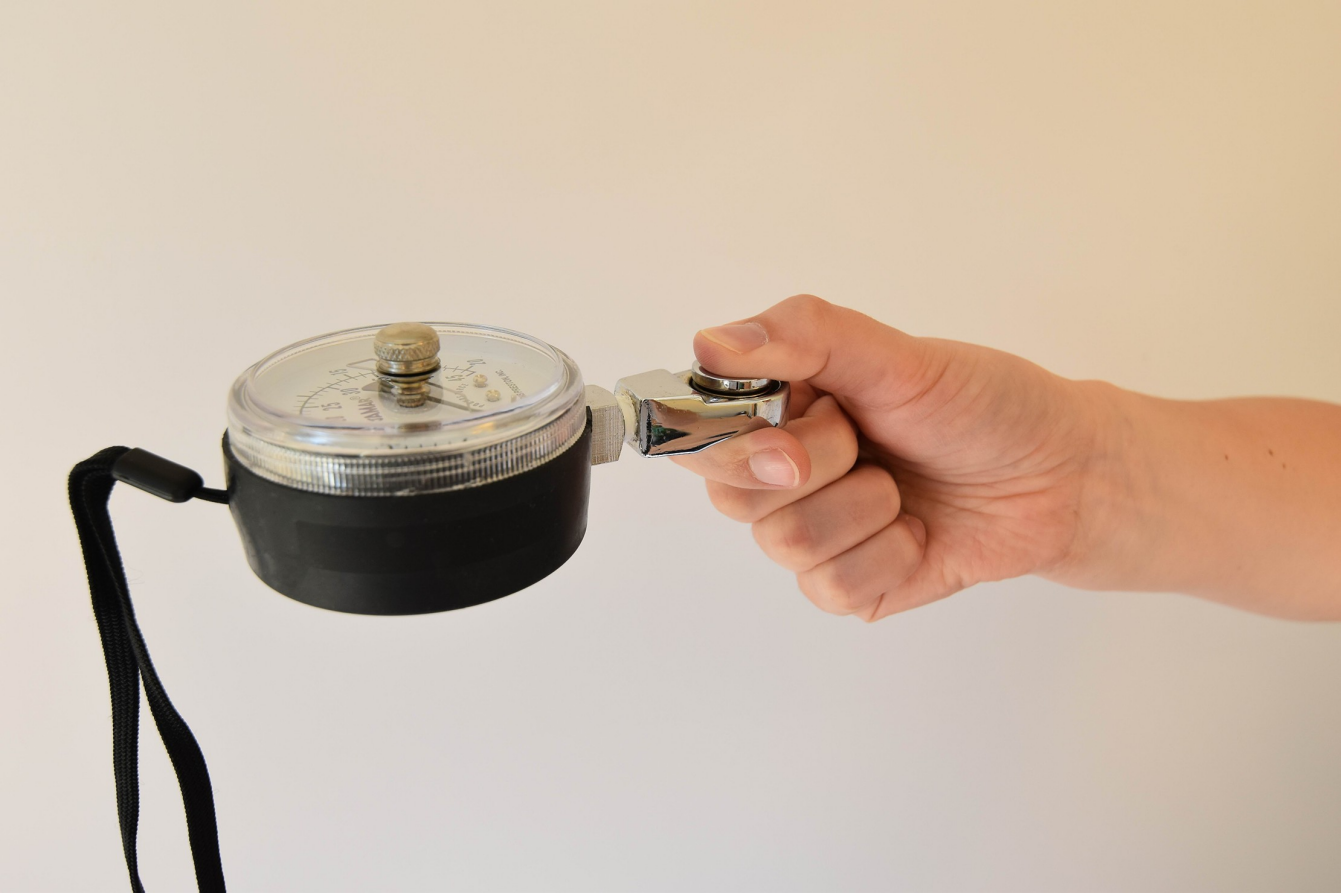
Photo of key grip grasp.

**Fig 3:**
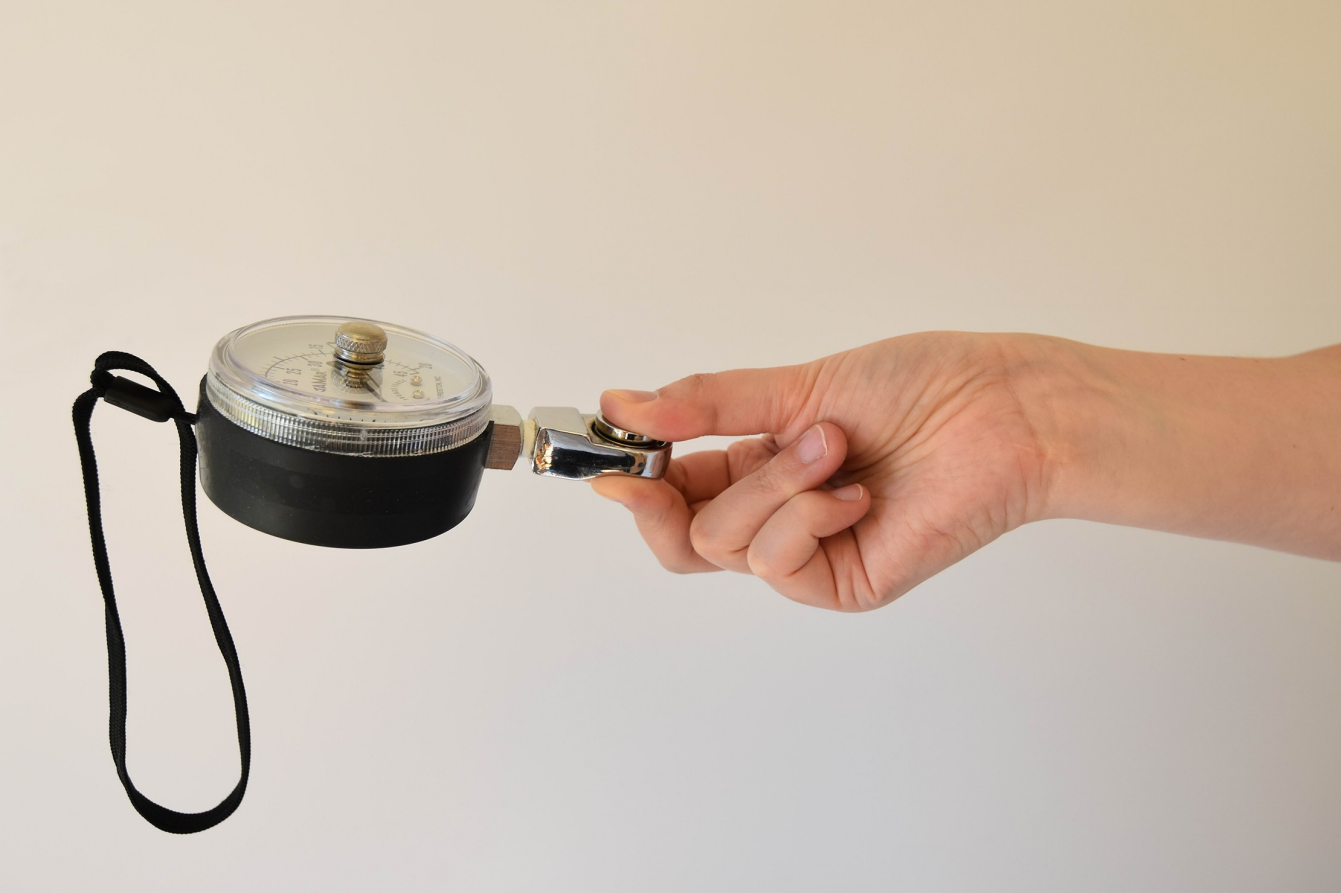
Photo of three-jaw chuck grasp.

General characteristics of the participants such as age, gender and hand dominance were registered. Details obtained on the fracture comprised location, type, (post) treatment, cast duration and potential complications. Grip strength was measured with the Jamar® hydraulic hand dynamometer (Lafayette Instrument Company, Lafayette, IN, USA). Participants were positioned according to the standardized testing position of the American Society of Hand Therapists (ASTH): seated subject, shoulders adducted and neutrally rotated, elbow flexed at 90°, wrist at 0–30° extension and 0–15° ulnar variation.[11] The handlebar was set to the second position for all participants, except children younger than 6 years, who because of their smaller hand size were tested at the first position. Strength of key grip (or lateral grasp) and three-jaw chuck grip were measured with the Jamar® hydraulic pinch gauge (Lafayette Instrument Company, Lafayette, IN, USA). Figs 1–3 illustrate these grasps. During each session all three strength measurements were performed twice on each side, and all individual attempts were scored. Both devices were calibrated. Verbal encouragement was given to encourage participants to try their best. Participants were asked if they experienced pain, and if so, whether they could rate it using a numeric rating scale (NRS) ranging from 0 (no pain) to 10 (worst pain imaginable). For those who found this to be difficult a Faces Scale was used, which is based on the same principle as a visual analogue scale but uses smileys. [12,13] Hand dominance was determined by asking which hand was used to write, or in the case of 4- and 5-year-olds which hand was used to draw a shape.

### Statistical analysis

Descriptive statistics were used to describe the main characteristics of the study population. For strength measurements the mean of the two attempts (grip, key or three-jaw chuck) of each hand was used in the analyses. To correct grip strength for the influence of hand dominance, the score of the affected hand was also compared to a calculated expected value of that hand (as if it were unaffected). This calculated value was derived from the adjusted scores of the unaffected hand according to findings from an earlier study by the current research group.[14] Scores between hands were compared for each measurement session and further by type of treatment using the Wilcoxon signed rank test.

To examine in more detail if there were differences on pattern of recovery between children who underwent a different treatment, a mixed-model repeated measurements analysis was performed for possible confounders (age, gender, affected dominant hand, fracture type). Variables noteworthy of altering the -2 restricted log likelihood of grip strength were ultimately taken into the final model.

Finally, multivariate linear regression analyses were performed to establish if the variables treatment type, fracture type, cast immobilization, occurrence of unwanted events (re-displacement or complication) and degree of pain were associated with an increase in the ratio between affected grip strength and expected strength. To this end, a ratio variable was created by dividing the affected value by the previously mentioned calculated expected value at all three measurement points. Extent of strength increase was used as the dependent variable and was defined as the difference in this ratio variable between measurement sessions (T2 minus T1 and T3 minus T2). In these analyses, pain was defined as occurrence of pain at 6 weeks or 3 months after trauma respectively. Results were considered to be significant when the associated p-value was <0.05. Statistical procedures were conducted using SPSS 23.0 for Windows (IBM SPSS Inc.).

## Results

### Demographic characteristics

**Fig 4:**
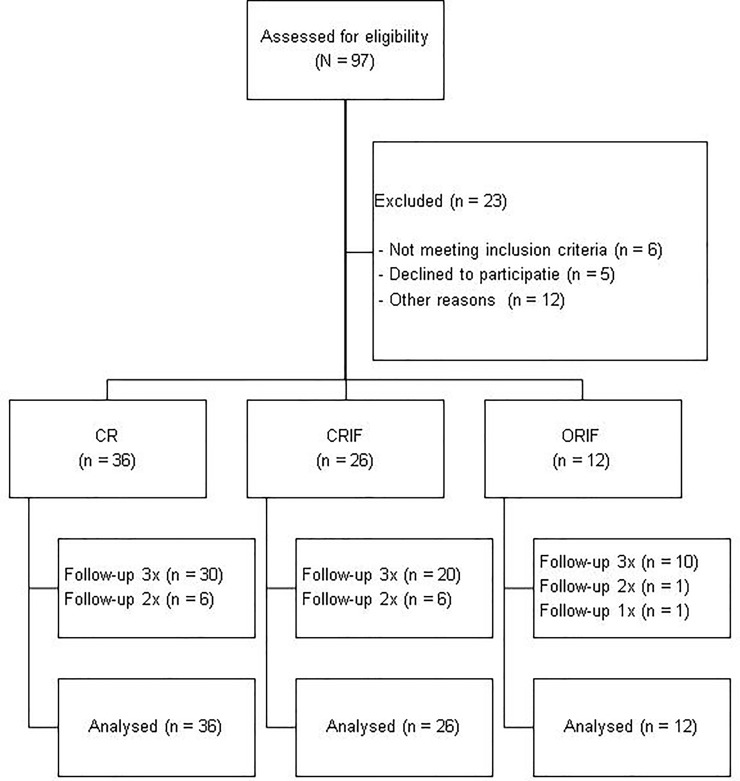
Enrollment flow diagram.

During the study period 97 children underwent an open or closed reduction of their fracture. Twenty children could not participate, 6 failed to meet criteria for inclusion, and another 14 could not be included due to other reasons (3 children had too extensive injuries, 5 families were not willing to participate, 4 children received follow-up in another hospital, and 2 families could not be reached for follow-up). Bilateral fractures occurred in 7.8% (N = 6) of children, all right-dominant. In 3 cases both fractures required repositioning and thus met criteria for inclusion. Since analyzing these participants twice could induce dependency within the data, they were excluded. The final study population therefore comprised 74 participants. An enrollment flow diagram is shown in Fig 4. The average age at which the fracture was sustained was 11.0 years (SD 3.6). The youngest participant was 4.6 years old, the oldest 17.5. Right-hand dominance was seen in 83.8% of the study population. Among the right-handed children a minority of 35.5% sustained a unilateral fracture on their dominant side, whereas in most left-handed children the dominant side was fractured, namely 66.7% of cases. A more detailed overview of the study population can be found in Table 1.

**Table 1:** Characteristics of the study population.

		*Total*	*Both-bone*	*Radius*	*Metacarpal*	*Phalanx*
*N*		74	37	17	9	11
*Mean age (SD)*		11.0 (3.7)	9.0 (3.2)	11.8 (3.3)	14.3 (4.0)	10.9 (3.4)
*Male gender (%)*		53 (71.6)	23 (62.2)	16 (94.1)	6 (66.7)	8 (72.7)
*Right-dominant (%)*		62 (83.8)	30 (81.1)	14 (82.4)	9 (100.0)	9 (81.8)
*Dominant side affected (%)*		29 (39.2)	14 (37.8)	9 (52.9)	5 (55.6)	3 (27.3)
*Treatment (%)*	CR	36 (48.6)	10 (27.0)	12 (70.6)	7 (77.8)	7 (63.6)
	CRIF	26 (35.1)	20 (54.1)	3 (17.6)	2 (22.2)	1 (9.1)
	ORIF	12 (16.2)	7 (18.9)	2 (11.8)		3 (27.3)

Calendar age at the time the fracture was sustained.

CR = closed reduction, CRIF = closed reduction internal fixation, ORIF = open reduction internal fixation.

In 16 participants an unwanted event occurred, either secondary dislocation or the endurance of a complication. In 10 participants angulation or rotation either did not improve or worsened, for which a secondary repositioning was performed. Complications were related to problems with Kirschner wires, imminent malunion or child factors (e.g. second trauma during treatment). Slightly more than half of the study population (53%) was pain-free within 6 weeks of trauma versus 76% at 3 months and 6 months after trauma. None of the participants experienced continuous pain–only in specific situations–and more importantly, none experienced pain while performing the measurements in this study.

### Grip strength

For all participants with a unilateral fracture, grip strength of the affected hand was compared to that of the unaffected hand at 3 measurement sessions. Overall, loss of strength amounted to 32.3% at 6 weeks, 12.8% at 3 months and 4.7% 6 months after trauma. This was analyzed further by type of treatment. The average loss of strength amounted to 24.1%, 6.8%, and -0.2% for fractures that were treated by CR, versus 42.3%, 15.9%, and 4.9% respectively for fractures treated by CRIF. Finally, loss of strength for fractures treated by ORIF was more prominent, amounting to 37.3%, 20.0% and 10.2%. Results showed a significant difference between the strength of the affected and unaffected hand for all types of treatments at 6 weeks and 3 months after trauma. However, after 6 months only the ORIF group still showed a significant strength difference between the hands. An overview of these results can be found in Table 2.

**Table 2:** Grip strength of the affected versus the unaffected hand by type of treatment.

		*Affected hand*					*Unaffected hand*				
		N	Mean (kg)	SD	Min	Max	Mean (kg)	SD	Min	Max	Sig. (2-tailed)
*T1*	Group	66	15.1	9.4	2.0	49.0	22.4	10.2	4.0	48.5	**<0.001**
	CR	32	17.5	9.8	3.5	49.0	22.5	9.6	8.0	44.0	**<0.001**
	CRIF	23	10.3	6.4	2.0	28.5	19.7	10.3	4.0	39.0	**<0.001**
	ORIF	11	18.4	10.2	3.5	35.5	27.7	10.6	17.0	48.5	**0.003**
*T2*	Group	69	20.1	10.5	3.5	56.0	23.0	11.2	5.5	54.5	**<0.001**
	CR	33	23.3	12.0	8.5	56.0	24.8	11.7	9.5	54.5	**0.008**
	CRIF	25	15.3	7.1	3.5	31.0	19.0	10.0	5.5	42.0	**0.001**
	ORIF	11	21.2	8.3	10.0	41.0	26.7	10.6	12.5	54.5	**0.004**
*T3*	Group	63	23.1	10.0	6.5	53.5	24.2	10.8	8.0	54.5	**0.011**
	CR	31	26.1	11.1	8.0	53.5	26.3	11.3	8.0	54.5	0.507
	CRIF	21	18.1	7.8	6.5	32.0	19.6	9.9	10.0	49.5	0.161
	ORIF	11	24.0	6.4	16.0	37.0	27.2	8.3	15.5	45.5	**0.020**

CR = closed reduction, CRIF = closed reduction internal fixation, ORIF = open reduction internal fixation.

To correct for the influence of hand dominance, grip strength of the affected hand was further compared to that of the calculated expected value, which was derived from the scores of the unaffected hand as described in the Methods section. This analysis did not lead to any changes in significance compared to the results as shown in Table 2.

### Key grip

Overall loss of strength was 22.0% at 6 weeks, 6.9% at 3 months and 1.8% at 6 months after trauma. For fractures treated by CR a significant loss of strength in key grip could only be observed at T1 (12.5%). Loss of strength after sustainment of fractures treated by CRIF and ORIF at 6 weeks was more prominent, 30.6% and 32.0% respectively, decreasing to 14.4% and 13.8% at 3 months. In both groups this difference was significant. Six months after sustainment of the fracture, loss of strength for the ORIF group was still 13.5%. An overview of these results can be found in Table 3.

**Table 3:** Key grip strength of the affected versus the unaffected hand by type of treatment.

		*Affected hand*					*Unaffected hand*				
		N	Mean (kg)	SD	Min	Max	Mean (kg)	SD	Min	Max	Sig. (2-tailed)
*T1*	Group	67	3.3	1.9	0.3	10.4	4.2	2.0	1.3	9.5	**<0.001**
	CR	32	3.8	2.0	0.9	10.4	4.3	1.9	1.4	9.0	**0.002**
	CRIF	24	2.5	1.5	0.5	6.6	3.6	1.9	1.3	8.5	**<0.001**
	ORIF	11	3.6	1.5	0.3	6.1	5.3	2.2	2.5	9.5	**0.005**
*T2*	Group	68	4.2	1.9	0.5	9.8	4.5	1.9	1.0	9.8	**0.001**
	CR	33	4.5	2.1	1.0	9.8	4.5	1.8	1.0	8.1	0.549
	CRIF	24	3.4	1.7	0.5	7.4	4.0	1.9	1.4	8.8	**0.001**
	ORIF	11	4.7	1.4	2.6	7.5	5.5	2.1	2.1	9.8	**0.032**
*T3*	Group	61	4.6	2.0	1.3	11.4	4.7	2.1	1.5	11.0	0.360
	CR	30	5.3	2.1	1.5	11.4	5.1	2.2	1.5	11.0	0.309
	CRIF	21	3.7	1.7	1.3	7.4	3.8	1.7	1.5	8.0	0.294
	ORIF	10	4.9	1.4	2.9	7.3	5.6	1.8	2.4	9.1	**0.041**

CR = closed reduction, CRIF = closed reduction internal fixation, ORIF = open reduction internal fixation.

6–9

### Three-jaw chuck

Overall loss of strength amounted to 22.1% at 6 weeks, 4.7% at 3 months and 3.2% at 6 months after trauma. For both the CR and CRIF group a significant difference was limited to the 6-week measurement (17.7% and 33.1% respectively). By contrast, the ORIF group still showed a significant difference in strength at 3 months amounting to 14.5%. Six months after trauma no significant difference in strength could be observed in any of the groups. An overview of these results can be found in Table 4.

**Table 4:** Three-jaw chuck of the affected versus the unaffected hand by type of treatment.

		*Affected hand*					*Unaffected hand*				
		N	Mean (kg)	SD	Min	Max	Mean (kg)	SD	Min	Max	Sig. (2-tailed)
*T1*	Group	64	2.6	1.5	0.4	7.6	3.3	1.9	0.3	7.8	**<0.001**
	CR	32	2.9	1.7	0.5	7.6	3.6	1.7	1.0	7.4	**<0.001**
	CRIF	22	2.0	1.3	0.3	5.3	3.0	2.0	0.3	7.8	**0.013**
	ORIF	10	2.7	1.4	1.3	6.1	4.0	1.9	1.3	7.3	**0.008**
*T2*	Group	68	3.4	1.8	0.5	8.9	3.6	1.9	0.6	9.0	0.082
	CR	33	3.8	2.2	0.9	8.9	3.8	2.0	0.9	9.0	0.836
	CRIF	24	2.8	1.3	0.5	5.0	3.0	1.7	0.6	5.9	0.109
	ORIF	11	3.5	1.8	1.8	7.8	4.1	2.1	1.8	9.0	**0.018**
*T3*	Group	61	4.0	1.7	1.1	9.1	4.2	1.8	1.3	8.6	0.401
	CR	30	4.7	1.8	1.4	9.1	4.8	1.9	1.8	8.6	0.705
	CRIF	21	3.1	1.5	1.1	5.9	3.1	1.4	1.3	6.8	0.951
	ORIF	10	4.0	1.0	2.4	5.4	4.6	1.3	2.3	6.3	0.155

CR = closed reduction, CRIF = closed reduction internal fixation, ORIF = open reduction internal fixation.

### Pattern of recovery of the affected hand between children who underwent a different treatment

A mixed-model repeated measurements analysis was performed to examine for differences in the pattern of strength recovery of the affected hand over time between participants who underwent different type of treatments (treatment x time). Time, age and gender were found to be of significant influence on all 3 grasps, and were therefore incorporated in the overall model. The dominant hand being affected and location of fracture were not of significant influence on strength recovery of the affected hand, hence removed from the model. Final results showed no difference in the pattern of recovery of the affected hand for any of the grasps over time between participants who received a different treatment. An overview of the p-values of this analysis of can be found in Table 5. Plots for the pattern of recovery for all three grasps can be found in the Supporting information (S1–S3 Figs).

**Table 5:** P-values of variables associated with strength recovery of the affected hand for the different grasps over time.

	*Grip*	*Key*	*Three-jaw chuck*
*Intercept*	**0.007**	0.057	0.454
*Treatment*	**0.042**	0.211	**0.011**
*Time*	**<0.001**	**<0.001**	**<0.001**
*Gender*	**0.001**	**0.009**	**<0.001**
*Age*	**<0.001**	**<0.001**	**<0.001**
*Treatment x time*	0.161	0.161	0.993

There is no significant difference in the pattern of recovery of the affected hand over time between participants undergoing different treatments (treatment x time)

### Factors associated with an increase in the ratio between affected grip strength and expected strength

Multivariate linear regression analyses were performed to establish which variables were associated with an increase in the ratio between affected grip strength and expected strength between the different measurement sessions. A larger ratio difference implies a larger strength increase towards ones expected (unaffected) strength during this timeframe, however not necessarily a better recovery as children with a larger delta could simply be worse off at the start of the timeframe. In the period of 6 weeks to 3 months female gender, type of fracture sustained (both-bone) and occurrence of an unwanted event showed to be significantly associated with a larger ratio difference. In the 3-6-month period the occurrence of an unwanted event still was associated with the increase in this ratio difference, whereas the same did no longer hold true for gender and type of fracture sustained. An overview of the p-values of these results can be found in Table 6. More detailed results from the performed analysis can be found in the Supporting information (S1 Table).

**Table 6:** P-values of variables associated with an increased ratio between affected grip strength and expected strength between 6 weeks and 3 months post-trauma (T1 to T2) and 3 months and 6 months post trauma (T2 to T3).

	*T1-T2*	*T2-T3*
*Intercept*	0.052	0.096
*Gender*	**0.021**	0.802
*Fracture type*	**0.019**	0.115
*Cast*	0.163	0.545
*Unwanted event*	**0.038**	**0.009**
*Age*	0.876	0.833
*Pain*	0.607	0.962

## Discussion

To our knowledge, this is the first study to prospectively focus on how strength recovers after reduced fractures of the forearm, wrist or hand in children. Results showed that loss of strength as compared to the value of the unaffected hand was more prominent and prolonged the more invasive the course of treatment, i.e. most extensive in the group receiving ORIF and least extensive in the group receiving CR only. In participants treated by CR, grip strength was significantly impaired up to 3 months after trauma whereas key grip and three-jaw chuck grip recovered within this period. Grip strength was similarly impaired in children treated by CRIF. Key grip was also still impaired in this group 3 months after trauma. In participants treated by ORIF, both grip strength and key grip were still significantly impaired 6 months after sustaining the fracture. Also, the three-jaw-chuck grip was impaired prolongedly compared to the other groups–up to 3 months. There was however no difference in pattern of recovery between the groups, all following a similar trend. Time passed since sustainment of the fracture, age and gender were of significant influence on the strength of the affected hand over time. The increase in ratio between the affected grip strength and expected strength between 6 weeks and 3 months was associated with female gender, type of fracture sustained (both-bone) and occurrence of an unwanted event. The difference is due to this ratio being lower at the beginning of this timeframe for participants who sustained a both-bone fracture or endured an unwanted event (they were more affected at the start). Between 3 and 6 months after trauma only the occurrence of an unwanted event was still significantly associated with an increase in this ratio. Although around 25% of participants still experienced pain both 3 months and 6 months after trauma, no association between pain score and ratio between affected and expected strength was found. This is most likely because none of the participants experienced pain performing the strength measurements. The presence of pain has thus not influenced the outcome of the strength measurements, but should nonetheless not be ignored as it concerns a substantial amount of children and could very well affect other (more prolonged or intensive) activities that fall beyond the scope of the current study.

Comparison to previous literature is difficult because studies taking strength measurements into account are scarce. Roth et al. (2014) evaluated functional outcome after manipulation of previously reduced re-displaced forearm fractures versus conservative treatment (no secondary manipulation) 1–8 years post-injury.[7] The study population was thus comparable to our CR group. Their study concluded that limitation of grip strength was minimal in both groups (3 kg in the re-manipulated and 1 kg in non-re-manipulated group). The CR group in the current study concurrently showed a limitation of 0.2 kg 6 months post-trauma. During a long-term follow-up Valencia et al. (2015) evaluated grip as well as pinch strength in 16 children who sustained nerve injuries due to a supracondylar fracture.[15] They found significant loss of strength for both grip and pinch strength on the injured side, yet in 81% of cases the injured side corresponded with the non-dominant hand, which might have negatively influenced these results. Cramer et al. (1992) compared grip strength in children treated either by closed reduction and percutaneous pinning or open reduction and percutaneous pinning in 29 cases of displaced supracondylar humeral fractures.[16] They calculated strength ratios (non-dominant/dominant strength) and found an average of 0.86 and 0.87 in children who injured their dominant or non-dominant extremity respectively. Comparisons of the current scores to both Valencia et al. (2015) and Cramer et al. (1992) would be inaccurate though, as these studies focus on an entirely different type of injury.[15,16]Yung et al. (2004) evaluated grip strength in displaced diaphyseal forearm fractures on average 70 months post-trauma.[4] In 76% of participants the grip strength of the affected hand was at least 95% that of the unaffected hand. The other 24% of participants scored between 70% and 90%. By comparison, in the current study this amounted to 43.9% and 29.8% respectively of participants with a radius or both-bone fracture 6 months after trauma. However, all these studies evaluated grip strength as an end result more than 1 year after trauma. Hence they offer no insight into recovery of strength during the initial months after trauma, whereas this is the focus of the current study. The same holds true for the study of Pershad et al. (2000), since it evaluated grip strength at the time of initial trauma only.[4]

Sinikumpu et al. 2013 also evaluated grip strength as an end result 9 to 14 years post-trauma. This was the only study using a control group to compare strength after sustainment of forearm shaft fractures in childhood.[17]No significant difference in grip strength was found between patients (mean 43.9 kg) and controls (mean 43.9). Boutis et al. (2010) compared grip strength of the affected hand in children with a minimally angulated distal radius fracture and found no difference between the cast and the splint group, although no comparison with the unaffected hand was made.[5] Davison et al. (2016) measured grip strength at 3, 6 and 12 weeks after sustainment of a fifth metacarpal neck fracture, finding decreased grip strength at 3 weeks (mean 10.5 kg) and 6 weeks (mean 3.8 kg) post-trauma in the ulnar gutter splint group and no significant differences (mean -0.6 kg) 12 weeks post-trauma.[10] In the current study average loss of strength for all metacarpal fractures at 6 weeks and 3 months amounted to 6.1 kg and 3.1 kg respectively. This might suggest that the fifth digit contributes less to grip strength than the other digits, but might also be the result of an age difference between the two studies.

A strong point of the current study was that besides grip strength, other standardized strength measurements often used by hand therapists–namely key grip and three-jaw chuck–were evaluated. All measurements were obtained at set moments in time corresponding to usual follow-up appointments. The follow-up rate was very high, with only one child withdrawing from further follow-up after the first measurement session. The lowest percentage of children completing a grip measurement session during the entire study period was 91.0%, for key grip and three-jaw grip at 6 months. A limitation of the current study was the heterogeneity of the study population itself, namely a large variance in age, type of fracture and type of treatment. This is why even though the study population was rather substantial to offer a first insight into the recovery of strength, subgroup analyses nonetheless quickly led to small groups. Future research should concentrate on a larger or less heterogenic study population. Also, pinch strength was unfortunately not evaluated even though it was initially intended. Researchers established that this specific measurement was difficult to perform on the smaller children and moreover that the set of measurements became too extensive to maintain the child’s interest. Pinch strength was therefore eliminated from the study protocol after the first measurement sessions.

The current study had a descriptive nature, so no treatment alterations were made. The fact that the ORIF group scored worse than the CRIF (and the CRIF worse than the CR) might simply be a reflection of the severity of the fracture sustained. Therefore, the current study will not have consequences for the management of pediatric forearm fractures. However, the relation between treatment invasiveness and the duration and severity of strength loss has to our knowledge not been described previously. This combined with the trend from conservative treatment toward surgical intervention for displaced fractures of the forearm calls for further research into this topic.[18–20] Ideally, a randomized controlled trial comparing recovery of strength between similar fractures (type, location and angulation) treated by means of different modalities should be conducted.

In conclusion, since the extent and duration of muscle strength loss for all strength measurements tend to be more prominent the more invasive the treatment chosen, as well as the fact that a large percentage of children still experience pain 6 months after trauma, referral to a hand therapist for additional guidance should be easily accessible to all children with a reduced fracture. In particular, referral should be considered when ORIF is chosen as the course of treatment.

## Supporting information

S1 Checklist TREND statement checklist. (PDF)

S1 Fig Plot for the pattern of recovery of grip strength of the affected hand. (TIF)

S2 Fig Plot for the pattern of recovery of key grip of the affected hand. (TIF)

S3 Fig Plot for the pattern of recovery of three-jaw chuck of the affected hand. (TIF)

S1 Table Parameter estimates from the multivariate linear regression establishing if the which variables were associated with an increase in the ratio between affected grip strength and expected strength for time period T1 to T2 and T2 to T3. (DOCX)

S1 Protocol Protocol for GOPRO study. (PDF)

S2 Protocol Protocol GOPRO studie. (PDF)

S1 Dataset (XLSX)
