# Supplementary figures and images for: Recovery of strength after reduced pediatric fractures of the forearm, wrist or hand; A prospective study
Source: PLoS One. 2020 Apr 1;15(4):e0230862. doi: 10.1371/journal.pone.0230862 (PMC7112181; doi:10.1371/journal.pone.0230862)

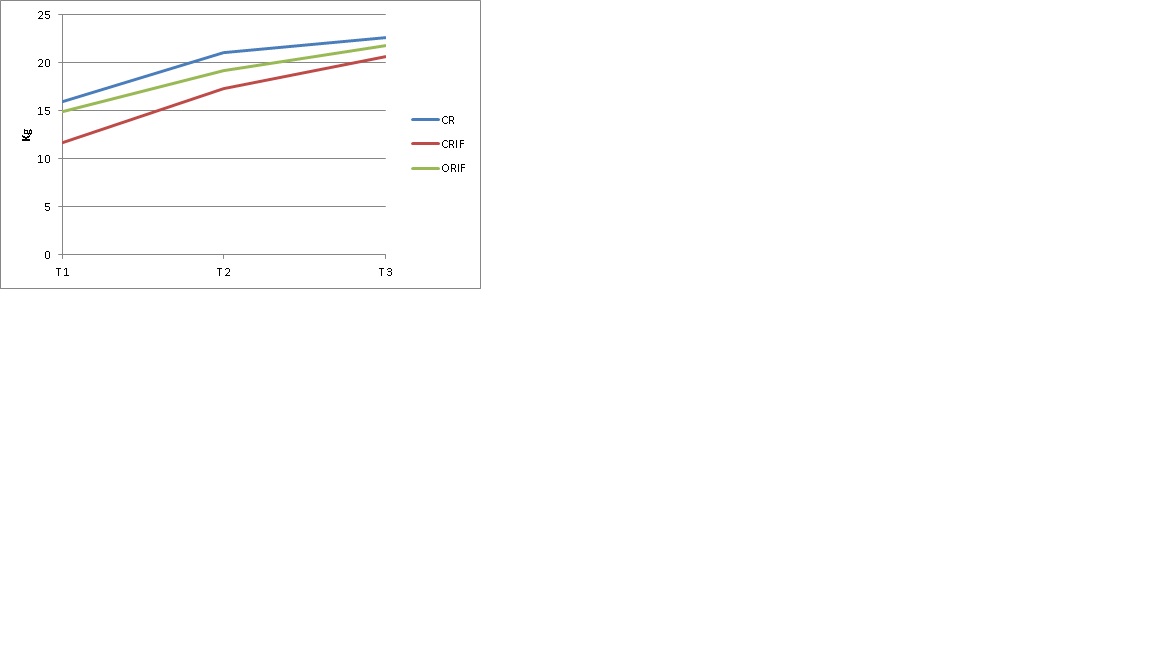

Supplement: S1 Fig — (TIF) [file pone.0230862.s002.tif]

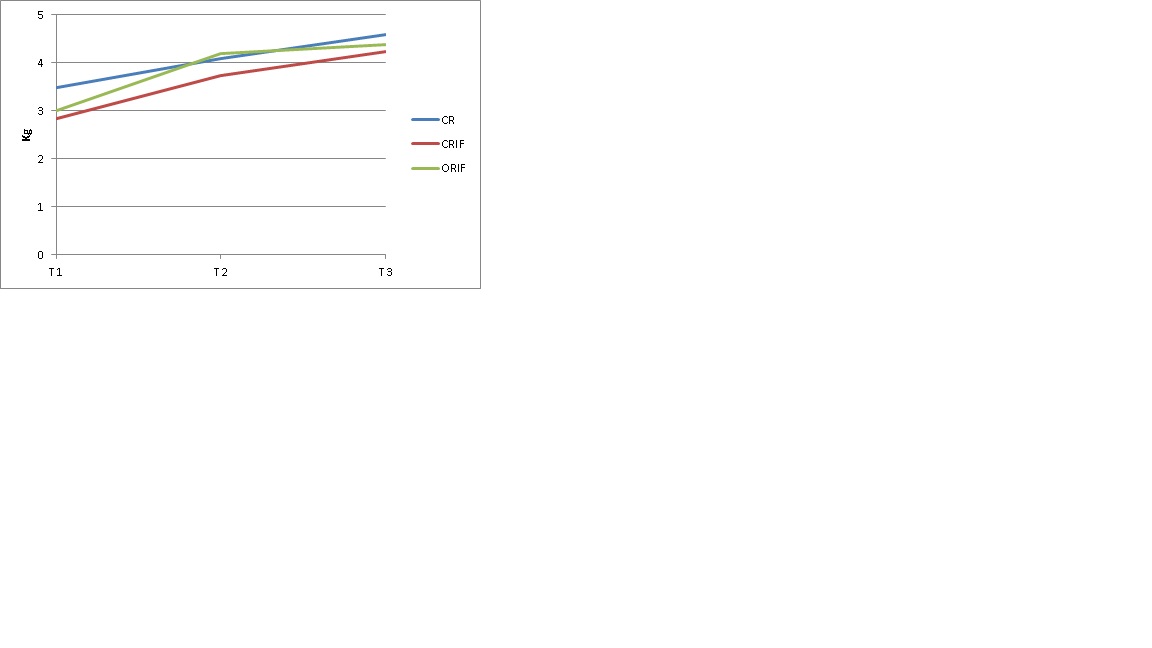

Supplement: S2 Fig — (TIF) [file pone.0230862.s003.tif]

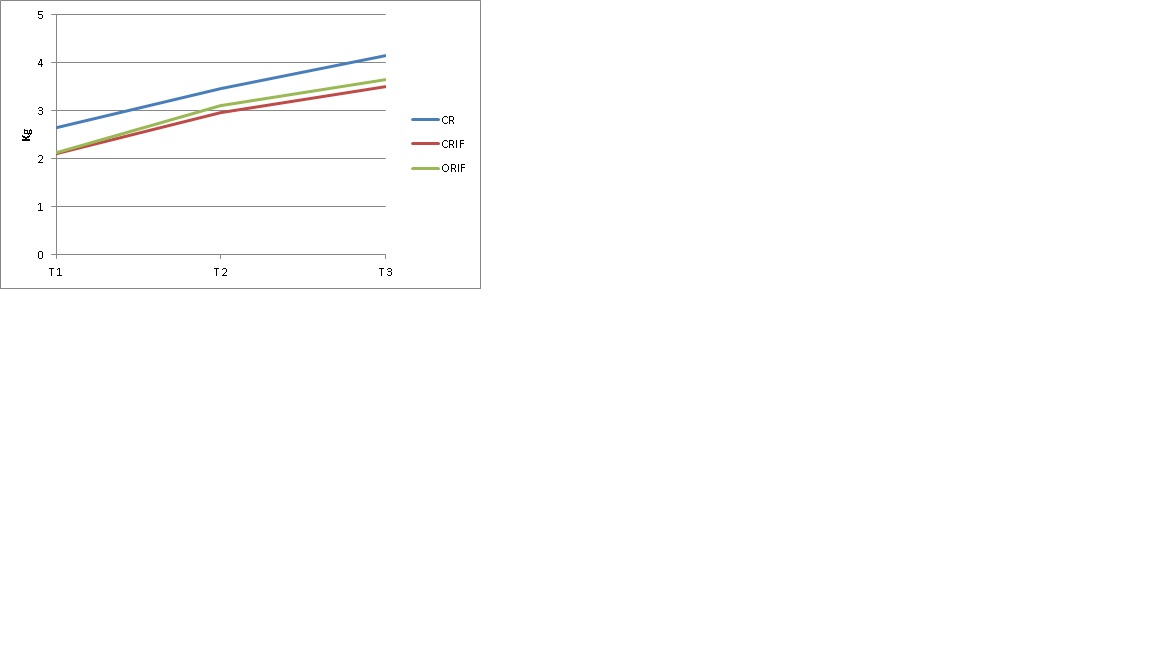

Supplement: S3 Fig — (TIF) [file pone.0230862.s004.tif]
