# Supplementary material for: Recovery of strength after reduced pediatric fractures of the forearm, wrist or hand; A prospective study
Source: PLoS One. 2020 Apr 1;15(4):e0230862. doi: 10.1371/journal.pone.0230862 (PMC7112181; doi:10.1371/journal.pone.0230862)
Supplement: S1 Table — (DOCX) [file pone.0230862.s005.docx]

S4 Tab. Parameter estimates from the multivariate linear regression establishing if the which variables were associated with an increase in the ratio between affected grip strength and expected strength for time period T1 to T2 and T2 to T3.

|  |  |  |  | T1 to T2  95% Wald CI | |  |  |  | T2 To T3  95% Wald CI | |  |
| --- | --- | --- | --- | --- | --- | --- | --- | --- | --- | --- | --- |
| Parameter |  | B | Std. Error | Upper | Lower | Sig. | B | Std. Error | Lower | Upper | Sig |
| Intercept |  | 0.290 | 0.1116 | 0.071 | 0.509 | 0.009 | 0.185 | 0.0997 | -0.011 | 0.380 | 0.064 |
| Gender | Male | -0.119 | 0.0513 | -0.219 | -0.018 | 0.021 | -0.012 | 0.0466 | -0.103 | 0.080 | 0.802 |
|  | Female | 0^a^ |  |  |  |  | 0^a^ |  |  |  |  |
| Fracture | Both-bone | 0.163 | 0.0660 | 0.034 | 0.292 | 0.013 | 0.098 | 0.0625 | -0.024 | 0.221 | 0.116 |
|  | Radius | 0.079 | 0.0761 | -0.071 | 0.228 | 0.302 | -0.021 | 0.0668 | -0.152 | 0.110 | 0.751 |
|  | Metacarpal | -0.020 | 0.0887 | -0.194 | 0.154 | 0.819 | 0.093 | 0.0824 | -0.068 | 0.255 | 0.257 |
|  | Carpal | 0^a^ |  |  |  |  | 0^a^ |  |  |  |  |
| Cast | No | -0.094 | 0.0673 | -0.226 | 0.038 | 0.163 | -0.035 | 0.0585 | -0.150 | 0.079 | 0.545 |
|  | Yes | 0^a^ |  |  |  |  | 0^a^ |  |  |  |  |
| Unwanted event | No | -0.126 | 0.0605 | -0.244 | -0.007 | 0.038 | -0.137 | 0.0523 | -0.239 | -0.034 | 0.009 |
|  | Yes | 0^a^ |  |  |  |  | 0^a^ |  |  |  |  |
| Age |  | 0.001 | 0.0068 | -0.012 | 0.014 | 0.876 | -0.001 | 0.0060 | -0.013 | 0.010 | 0.833 |
| Pain |  | 0.007 | 0.0143 | -0.021 | 0.035 | 0.607 | -0.001 | 0.0137 | -0.028 | 0.026 | 0.962 |
| (Scale) |  | ,030^b^ | 0.0053 | 0.021 | 0.042 |  | ,022^b^ | 0.0041 | 0.016 | 0.032 |  |
|  |  |  |  |  |  |  |  |  |  |  |  |
|  |  |  |  |  |  |  |  |  |  |  |  |
|  |  |  |  |  |  |  |  |  |  |  |  |
