# Supplementary material for: Recovery of strength after reduced pediatric fractures of the forearm, wrist or hand; A prospective study
Source: PLoS One. 2020 Apr 1;15(4):e0230862. doi: 10.1371/journal.pone.0230862 (PMC7112181; doi:10.1371/journal.pone.0230862)
Supplement: S1 Protocol — (PDF) [file pone.0230862.s006.pdf]

Protocol for GOPRO study

Objective of the study

The Departments of Orthopedics, Traumatology and Rehabilitation Medicine have developed a study to follow up minors who have had a fracture of the hand, wrist or lower arm. The objective of the study is to achieve better follow-up of the recovery and obtain more insight into any limitations and/or complaints in the long term. The study aims at all children whose fracture had to be reset or operated.

Procedure

Within 4 weeks of incurring the fracture, the patient and the parents will be approached by the treating physician, plaster physician or researcher to participate in the study. If they have other questions, an appointment can already be made at that point with the researcher, for example combined with a change of plaster. If they decide to participate, parents or guardians will be asked to sign a statement of consent. The child will subsequently have three appointments with the researcher: after 6 weeks, 3 months, and 6 months after incurring the fracture. These appointments are scheduled together with the regular follow-up appointments at the Traumatology or Orthopedics departments. If this is not possible, a house call will be offered. This makes it unnecessary for parents and child to have to make extra visits to the hospital.

What is measured

General data to be recorded include sex, age, the dominant hand (children under age 6 will be asked to draw a shape), the affected hand, and extensive details about the fracture and the treatment (see form). There will be questions about complaints of pain, stiffness, sensitivity problems and the like. Pain will be determined using an NRS scale. If this is still too difficult for the child, a Faces Scale can be used. Next, the researcher will find out whether there are issues such as redness, swelling or sensitivity problems. All measurements are first demonstrated to the child by the researcher, and tested first on the nonaffected side. It goes without saying that the parent/guardian will be present during this testing. In the first measurement the mobility of both the left and the right elbow, wrist and fingers will be accurately determined, and in subsequent measurements only the affected side.

- Baseline goniometer
  - Elbow flexion/extension, lateral olecranon as reference point.
  - Dorsal flexion/palmar flexion of wrist, over dorsal side, measuring arm between radius and ulna, measuring arm between MC II and III.
  - Radial/ulnar deviation, over dorsal side, lunate as reference point.
  - Abduction dig I, over dorsal side, lunate as a reference point.
- Baseline pronation/supination inclinometer
  - Sitting, shoulder in neutral position, elbow 90° degrees flexion.
- Baseline finger goniometer
  - Flexion/extension MCPs, IPs over dorsal side of the joints.

Next, the strength of the hand and fingers are determined (at all measuring moments bilaterally). Grip strength with the Jamar® hydraulic hand dynamometer according to the ASTH: sitting, shoulder in neutral position, elbow 90° flexion, wrist in 0-30° dorsal flexion and 0-15° ulnar deviation. For all children on handgrip position 2, except for children under the age of 6 years (handgrip position 1). Key pinch, tridigit pinch and tweezers test using the Jamar® hydraulic pinch gauge in the same starting position. The child is encouraged to do his/her best.

PATIENT INFORMATION

To participate in this scientific study:

Six-month follow-up of children with a fracture of the hand, wrist or lower arm treated by means of closed or open repositioning.

Dear parents/guardians,

<u>Objective of the study</u>

The Departments of Orthopedics, Traumatology and Rehabilitation Medicine have developed a study for more accurate follow-up of minors who have had a fracture of the hand, wrist or lower arm. The objective of the study is to achieve better follow-up of the recovery and obtain more insight into any limitations and/or complaints in the long term. The study aims at all children whose fracture had to be reset or operated.

<u>Procedure</u>

Within 4 weeks of incurring the fracture you will be approached by your treating physician, a plaster physician or a researcher to participate in the study. If you have other questions, an appointment can already be made at that point with the researcher, for example combined with a change of plaster. If you decide to participate, as parents or guardians you will be asked to sign a statement of consent. Your child will subsequently have three appointments with the researcher: on the day that the plaster is removed or the hand can be fully loaded again, after about 3 months, and after about 6 months. These appointments are scheduled together with the regular follow-up appointments at the Traumatology or Orthopedics departments. This makes it unnecessary to make extra visits to the hospital.

<u>What is determined/measured</u>

General data to be determined are the sex and age of your child, the dominant hand, and details about the fracture and treatment. The researcher will also ask your child whether he/she experiences symptoms of pain, stiffness, sensitivity problems and the like. Next, the researcher will find out whether there are issues such as redness, swelling or sensitivity problems, and will accurately measure the mobility of both the left and the right elbow, wrist and fingers. Lastly, the strength of the hand and fingers will be determined. All measurements are first demonstrated to the child by the researcher, and tested first on the nonaffected side. It goes without saying that you as parent/guardian will be present during this testing.

<u>Risks</u>

The Medical Ethics Testing Committee of UMCG has granted an exemption of the requirement to get insurance for this study. The reason is that the Committee considers that the nature of the study entails no risk for the participants.

<u>Confidentiality</u>

Access by qualified persons to data that can be traced back to your child can only be given with your authorization. These persons are staff of the research team, staff of the Health Care Inspectorate, and members of the Medical Ethics Testing Committee. Access may be necessary to assess the reliability and quality of the study. Research data will be used in compliance with the Personal Data Protection Act. Personal data that is collected during this study will be replaced by a code. Only that code will be used for study documentation, in reports and in publications about this study. Only those who have the key to the code (the researcher and/or the treating physician) know who the person behind the code is. The data is kept for 15 years.

STATEMENT OF CONSENT

To participate in this scientific study:

Six-month follow-up of children with a fracture of the hand, wrist or lower arm treated by means of closed or open repositioning.

FOR THE LEGAL REPRESENTATIVES

I have been asked to give authorization for the participation of

Name and initials:
Date of birth:

I have been satisfactorily informed about the study. I have read the written information thoroughly. I have been given the opportunity to ask questions about the study. My questions have been satisfactorily answered. I know that medical data relevant to this study will be used for scientific research and may be published. I consent to this as long as my privacy is protected.

We agree / I agree to the participation of the person listed above in this study.

Name and initials:
Relationship to the participant:

Signature: Date:

Name and initials:
Relationship to the participant:

Signature: Date:

The undersigned declares that the persons listed above have been informed orally as well as in writing about the study specified above. He/she also declares that prematurely ending the participation of this person will not affect the care he or she needs in any way whatsoever.

Name:
Position:

Signature: Date:

** This form is earmarked for research with minors. Authorization must be given by the legal representatives.*

First visit

ID: ________
Measurement: ________
Date: ________

Sex: Boy / Girl

Date of birth: ________

Age: ________ years

Dominant arm: Right / Left

Affected side: Right / Left

Cause ____

Date of fracture: ________, ________ days after fracture.

Type of fracture: ____

____

____

Angulation: ________________________________*(to be assessed by radiologist)*

Treatment: ____

____

____

Post-treatment: ____

____

____

Current situation: Plaster removal / Peak loading allowed / Other, specify:

____

Physiotherapy + / -

Pain: 0 – 1 – 2 – 3 – 4 – 5 – 6 – 7 – 8 – 9 – 10

Swelling: No / Yes, specify: ____

Redness: No / Yes, specify: ____

Allodynia No / Yes

Temperature asymmetric: No / Yes

Trophic features: No anomalies / Anomalies (specify further below)
Skin/ hair / nail growth / sweating pattern:

____

____

Sensitivity: Undisturbed / Disturbed, specify:

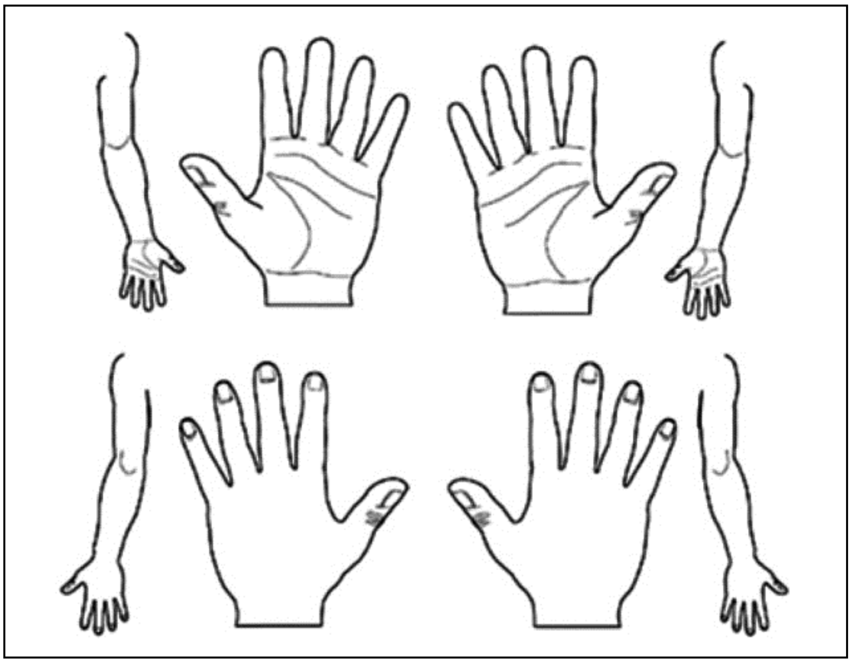

Locomotion: Nine-Hole Peg Test
Right: __________ seconds
Left: __________ seconds

**Elbow**	**Right**	**Left**	
Ex/Fl			

**Wrist**			
DFl/PFl			
Rdev/Udev			
Pro/Sup			

**Fingers**	**(Ex/Fl)**		
DIP-II			°
DIP-III			°
DIP-IV			°
DIP-V			°
PIP-II			°
PIP-III			°
PIP-IV			°
PIP-V			°
MCP-II			°
MCP-III			°
MCP-IV			°
MCP-V			°
IP			°
MCP-1			°
Lat Abd			°
Kapanji			1-10

**Strength**			
Grip 1			kg
Grip 2			kg
Pinch 1			kg
Pinch 2			kg
Key 1			kg
Key 2			kg
3JC 1			kg
3JC 2			kg

Follow-up visit

ID:

Measurement:

Date:

Complication:

Physiotherapy:

Current situation:

Researcher:

Experienced limitations:

Full use:

Pain:

Swelling:

Redness:

Allodynia:

Temperature asymmetrical:

Trophic features:

Sensitivity:

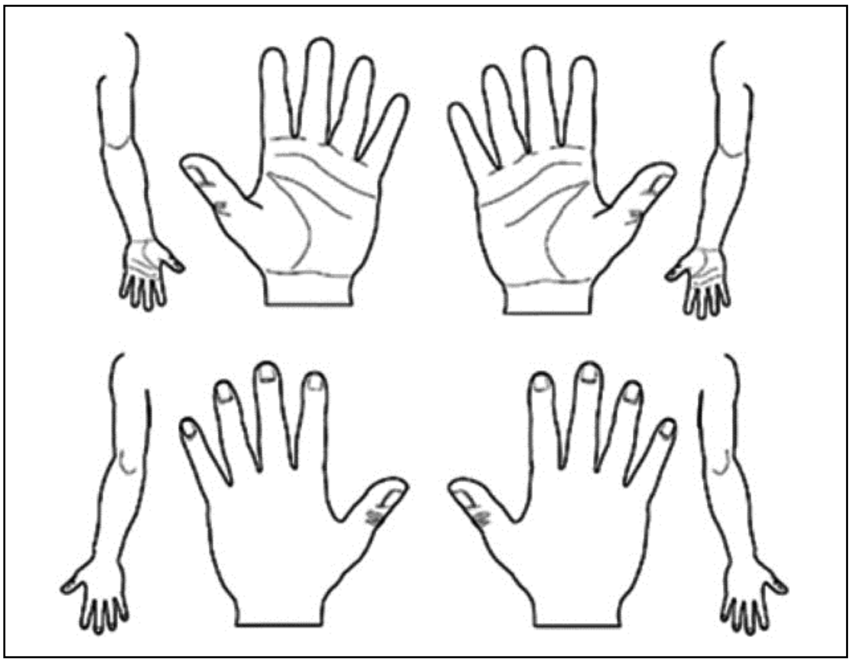

Locomotion: Nine-Hole Peg Test
Right:
Left:

**Elbow**	**Affected side**
Ex/Fl	____________
**Wrist**	
DFl/PFl	____________
Rdev/Udev	____________
Pro/Sup	____________
**Fingers**	**(Ex/Fl)**
DIP-II	____________
DIP-III	____________
DIP-IV	____________
DIP-V	____________
PIP-II	____________
PIP-III	____________
PIP-IV	____________
PIP-V	____________
MCP-II	____________
MCP-III	____________
MCP-IV	____________
MCP-V	____________
IP	____________
MCP-1	____________
Lat Abd	____________
Kapanji	____________

**Strength**	**Right**	**Left**	
Grip 1	____________	____________	kg
Grip 2	____________	____________	kg
Pinch 1	____________	____________	kg
Pinch 2	____________	____________	kg
Key 1	____________	____________	kg
Key 2	____________	____________	kg
3JC 1	____________	____________	kg
3JC 2	____________	____________	kg
