# Supplementary material for: Recovery of strength after reduced pediatric fractures of the forearm, wrist or hand; A prospective study
Source: PLoS One. 2020 Apr 1;15(4):e0230862. doi: 10.1371/journal.pone.0230862 (PMC7112181; doi:10.1371/journal.pone.0230862)
Supplement: S2 Protocol — (PDF) [file pone.0230862.s007.pdf]

Protocol GOPRO studie

Doel van het onderzoek

Vanuit de afdelingen orthopedie, traumatologie en revalidatiegeneeskunde is huidig onderzoek opgezet om minderjarigen die een breuk hebben opgelopen van hand, pols of onderarm nauwkeuriger te vervolgen. Doel van het onderzoek is om het herstel beter te kunnen vervolgen en meer zicht te krijgen op eventuele beperkingen en/of klachten op de lange termijn. Het onderzoek richt zich op alle kinderen waarbij de breuk gezet of geopereerd moest worden.

Procedure

In de periode tot 4 weken na het oplopen van de breuk worden patiënt en ouders benaderd door behandelend arts, gipsverbandmeester of onderzoeker om deel te nemen aan het ondezoek. Indien men verdere vragen heeft kan alvast van tevoren een afspraak worden gemaakt bij de onderzoeker, bijvoorbeeld in aansluiting op een gipswissel. Indien men besluit mee te doen, zal er van ouders of verzorgers gevraagd worden een toestemmingsverklaring te ondertekenen. Het kind zal hierna 3 afspraken krijgen bij de onderzoeker: na 6 weken, 3 maanden en 6 maanden na ontstaan van de fractuur. Deze afspraken worden aansluitend gepland aan de reguliere vervolgafspraken bij de afdeling traumatologie of orthopedie. Indien dit niet mogelijk is wordt een huisbezoek aangeboden. Extra bezoeken aan het ziekenhuis zijn voor ouders en kind hierdoor niet noodzakelijk.

Wat wordt gemeten

Algemene gegevens die worden vastgelegd omvatten geslacht, leeftijd, de dominante hand (bij kinderen onder de 6 een vorm laten tekenen), de aangedane hand en uitgebreide details over de breuk en behandeling (zie invulformulier). Gevraagd wordt naar klachten van pijn, stijfheid, gevoelsproblemen en dergelijke. Pijn wordt vastgelegd middels een NRS schaal. Als dit nog te lastig is voor het kind mag een Faces Scale gebruikt worden. De onderzoeker gaat vervolgens na of er sprake is van zaken als roodheid, zwelling of gevoelsproblemen. Alle metingen worden eerst door de onderzoeker aan het kind voorgedaan en eerst worden getest aan de niet-aangedane zijde. Vanzelfsprekend is de ouder/verzorger zelf bij dit onderzoek aanwezig. Bij de eerste meting zal van zowel de linker- als de rechter elleboog, pols en vingers de beweeglijkheid nauwkeurig bepaald worden, bij vervolgmetingen alleen de aangedane zijde.

- Baseline goniometer
  - Elleboog flexie/extensie, laterale olecranon als referentiepunt.
  - Dorsaalflexie/palmairflexie pols over dorsale zijde, meetarm tussen radius en ulna, meetarm tussen MC II en III.
  - Radiair/ulnairdeviatie, over dorsale zijde, lunatum als referentiepunt.
  - Abductie dig I, over dorsale zijde, lunatum als referentiepunt.
- Baseline pronation/supination inclonemeter
  - Zittend, schouder in neutrale positie, elleboog 90° graden flexie.
- Baseline finger goniometer
  - Flexie/extensie MCP’s, IP’s over dorale zijde van de gewrichten.

Voorts zal de kracht van de hand en vingers worden bepaald (op alle meetmomenten beiderzijds). Grijpkracht met de Jamar® hydraulic hand dynamometer volgens de ASTH: zittend, schouder in neutral positive, elleboog 90 graden flexie, pols in 0-30 graden dorsaalflexie en 0-15 graden ulnairdeviatie. Voor alle kinderen op handgreep stand 2, behoudens voor kinderen onder de 6 jaar (handgreep stand 1). Sleutel-, driepunt- en pincetgreep met de Jamar® hydraulic pinch gauge in zelfde uitgangspositie. Kind wordt aangemoedigd om zijn/haar best te doen.

PATIENTENINFORMATIE

Voor deelname aan het wetenschappelijk onderzoek:

Zes maanden follow-up van kinderen met een breuk van hand, pols of onderarm behandeld middels gesloten of open repositie.

Geachte ouders/verzorgers,

Doel van het onderzoek

Vanuit de afdelingen orthopedie, traumatologie en revalidatiegeneeskunde is een onderzoek opgezet om minderjarigen die een breuk hebben opgelopen van hand, pols of onderarm nauwkeuriger te vervolgen. Doel van het onderzoek is om het herstel beter te kunnen vervolgen en meer zicht te krijgen op eventuele beperkingen en/of klachten op de lange termijn. Het onderzoek richt zich op alle kinderen waarbij de breuk gezet of geopereerd moest worden.

Procedure

In de periode tot 4 weken na het oplopen van de breuk wordt u benaderd door uw behandelend arts, gipsverband-meester of onderzoeker om deel te nemen. Indien u verdere vragen heeft kan alvast van tevoren een afspraak worden gemaakt bij de onderzoeker, bijvoorbeeld in aansluiting op een gipswissel. Indien u besluit mee te doen, zal er van u als ouders of verzorgers gevraagd worden een toestemmingsverklaring te ondertekenen. Uw kind zal hierna 3 afspraken krijgen bij de onderzoeker: op de dag dat het gips verwijderd wordt of de hand weer volledig belast mag worden, na ongeveer 3 maanden en na ongeveer 6 maanden. Deze afspraken vallen samen met de reguliere vervolgafspraken bij de afdeling traumatologie of orthopedie. Extra bezoeken aan het ziekenhuis zijn hierdoor niet nodig.

Wat wordt bepaald/gemeten

Algemene gegevens die worden vastgelegd zijn het geslacht en de leeftijd van uw kind, de dominante hand, details over de breuk en behandeling. De onderzoeker zal uw kind ook vragen of hij/zij klachten ervaart van pijn, stijfheid, gevoelsproblemen en dergelijke. De onderzoeker gaat vervolgens na of er sprake is van zaken als roodheid, zwelling of gevoelsproblemen en zal van zowel de linker- als de rechter elleboog, pols en vingers de beweeglijkheid nauwkeurig meten. Tot slot zal de kracht van de hand en vingers worden bepaald. Alle onderzoeken zullen eerst door de onderzoeker aan het kind worden voorgedaan en eerst worden getest aan de niet-aangedane zijde. Vanzelfsprekend bent u als ouder/verzorger ook zelf bij dit onderzoek aanwezig.

Risico's

De Medisch-Ethische Toetsingscommissie van het UMCG heeft ontheffing verleend van de verplichting om voor dit onderzoek een verzekering af te sluiten. De reden hiervoor is dat de commissie van oordeel is dat dit onderzoek naar zijn aard voor de deelnemers eraan zonder enig risico is.

Vertrouwelijkheid gegevens

Tot uw kind herleidbare onderzoeksgegevens kunnen slechts met uw toestemming door daartoe bevoegde personen worden ingezien. Deze personen zijn medewerkers van het onderzoeksteam, medewerkers van de Inspectie voor de Gezondheidszorg en leden van de Medisch-Ethische Toetsingscommissie. Inzage kan nodig zijn om de betrouwbaarheid en kwaliteit van het onderzoek na te gaan. Onderzoeksgegevens zullen worden gehanteerd met inachtneming van de Wet bescherming persoonsgegevens. Persoonsgegevens die tijdens deze studie worden verzameld, zullen worden vervangen door een codenummer. Alleen dat nummer zal gebruikt worden voor studiedocumentatie, in rapporten of publicaties over dit onderzoek. Slechts degene, die de sleutel van de code heeft (de onderzoeker en/of behandelend arts) weet wie de persoon achter het codenummer is. De gegevens worden gedurende 15 jaar bewaard.

TOESTEMMINGSVERKLARING

Voor deelname aan het wetenschappelijk onderzoek:

Zes maanden follow-up van kinderen met een breuk van hand, pols of onderarm behandeld middels gesloten of open repositie.

<u>VOOR DE WETTELIJKE VERTEGENWOORDIGERS</u>

Mij is gevraagd om toestemming te verlenen voor deelname aan bovenvermeld onderzoek voor

Achternaam en voorletters:

Geboortedatum:

Ik ben naar tevredenheid over het onderzoek geïnformeerd. Ik heb de schriftelijke informatie goed gelezen. Ik ben in de gelegenheid gesteld om vragen over het onderzoek te stellen. Mijn vragen zijn naar tevredenheid beantwoord. Ik weet dat voor dit onderzoek relevante medische gegevens gebruikt worden voor wetenschappelijk onderzoek en eventueel gepubliceerd worden. Hiermee stem ik in mits privacy gewaarborgd wordt.

Wij stemmen / ik stem toe met deelname van bovenvermeld persoon aan het onderzoek

Achternaam en voorletters:

Relatie tot de deelnemer:

Handtekening: Datum:

Achternaam en voorletters:

Relatie tot de deelnemer:

Handtekening: Datum:

Ondergetekende verklaart dat de hierboven genoemde personen zowel mondeling als schriftelijk over het bovenvermelde onderzoek geïnformeerd zijn. Hij/zij verklaart tevens dat een voortijdige beëindiging van de deelname door bovengenoemde persoon, van geen enkele invloed zal zijn op de zorg die hem of haar toekomt.

Naam:

Functie:

Handtekening: Datum:

** Dit formulier is bestemd voor onderzoek met minderjarigen. Toestemming moet worden verleend door de wettelijke vertegenwoordigers.*

Eerste bezoek

ID: ________
Meting: ________
Datum: ________

Geslacht: Jongen / Meisje

Geboortedatum: ________

Leeftijd: ________ jaar

Dominante arm: Rechts / Links

Aangedane zijde: Rechts / Links

Oorzaak ____

Datum fractuur: ________, ________ dagen na fractuur.

Soort fractuur: ____

____

____

Angulatie: ______________________________*(te beoordelen door radioloog)*

Behandeling: ____

____

____

Nabehandeling: ____

____

____

Huidige situatie: Gipsverwijdering / Piekbelasting toegestaan / Anders nl:

____

Fysiotherapie + / -

Pijn: 0 – 1 – 2 – 3 – 4 – 5 – 6 – 7 – 8 – 9 – 10

Zwelling: Nee / Ja nl.: ____

Roodheid: Nee / Ja nl.: ____

Allodynie: Nee / Ja

Temperatuur asym: Nee / Ja

Trofische kenmerken: Geen afwijkingen / Afwijkingen (nader specificeren hieronder)
Huid / beharing / nagelgroei / zweetpatroon:

____

____

Sensibiliteit: Ongestoord / Gestoord nl.:

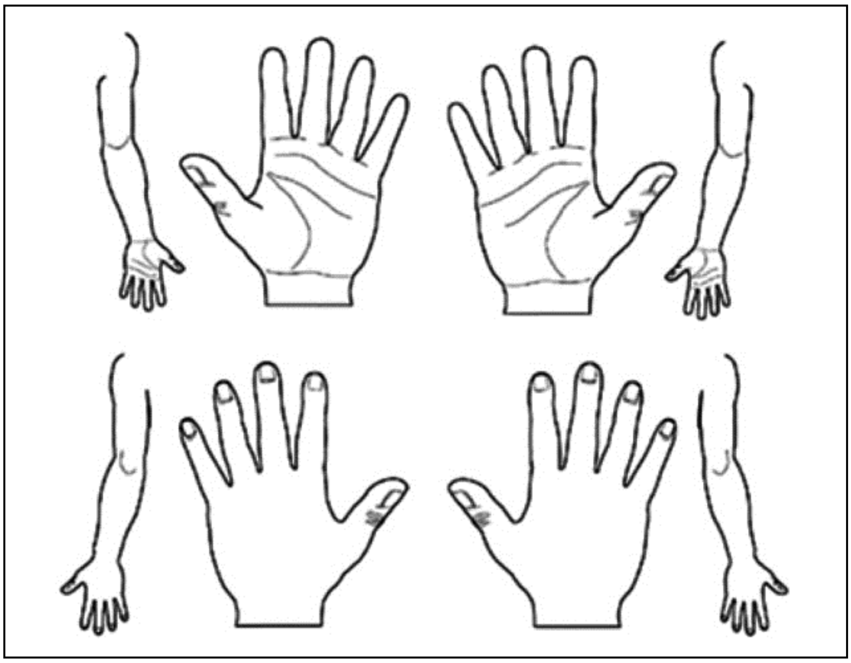

Motoriek: Nine-Hole Peg Test
Rechts: __________ seconden
Links: __________ seconden

	Rechts	Links	
**Elleboog**	**Rechts**	**Links**	
Ex/Fl	______	______	
**Pols**			
DFl/PFl	______	______	
Rdev/Udev	______	______	
Pro/Sup	______	______	
**Vingers**	**(Ex/Fl)**		
DIP-II	______	______	°
DIP-III	______	______	°
DIP-IV	______	______	°
DIP-V	______	______	°
PIP-II	______	______	°
PIP-III	______	______	°
PIP-IV	______	______	°
PIP-V	______	______	°
MCP-II	______	______	°
MCP-III	______	______	°
MCP-IV	______	______	°
MCP-V	______	______	°
	______	______	
IP			°
MCP-1	______	______	°
Lat Abd	______	______	°
Kapanji	______	______	1-10
**Kracht**			
Grip 1	______	______	kg
Grip 2	______	______	kg
Pinch 1	______	______	kg
Pinch 2	______	______	kg
Key 1	______	______	kg
Key 2	______	______	kg
3JC 1	______	______	kg
3JC 2	______	______	kg

Vervolgbezoek

ID:

Meting:

Datum:

Complicatie:

Fysiotherapie:

Huidige situatie:

Onderzoeker:

Zelf ervaren beperkingen:

Inzet volledig:

Pijn:

Zwelling:

Roodheid:

Allodynie:

Temperatuur asym:

Trofische kenmerken:

Sensibiliteit:

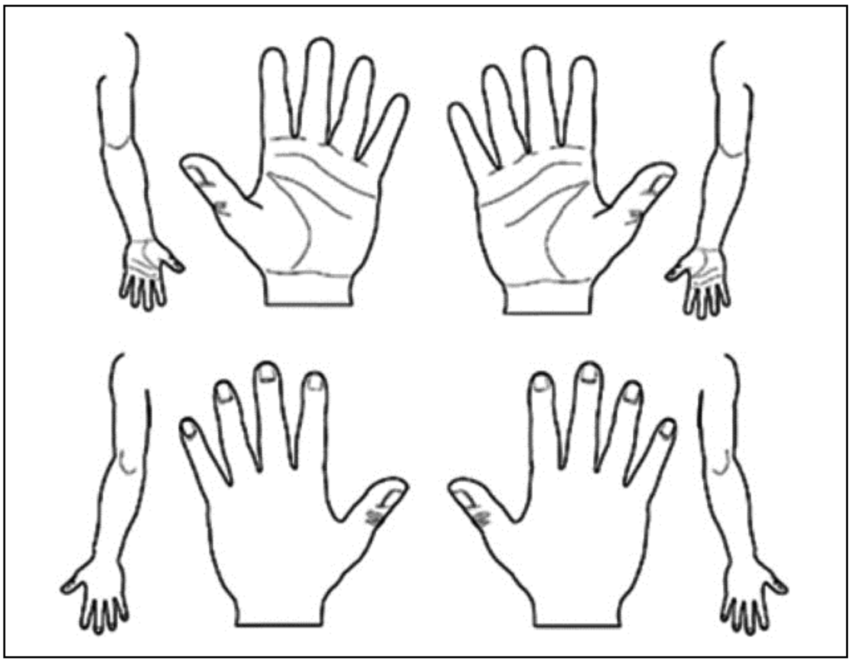

Motoriek: Nine-Hole Peg Test

Rechts:

Links:

**Elleboog**	**Aangedane zijde**
Ex/Fl	____________
**Pols**	
DFl/PFl	____________
Rdev/Udev	____________
Pro/Sup	____________
**Vingers**	**(Ex/Fl)**
DIP-II	____________
DIP-III	____________
DIP-IV	____________
DIP-V	____________
PIP-II	____________
PIP-III	____________
PIP-IV	____________
PIP-V	____________
MCP-II	____________
MCP-III	____________
MCP-IV	____________
MCP-V	____________
IP	____________
MCP-1	____________
Lat Abd	____________
Kapanji	____________

	**Rechts**	**Links**	
**Kracht**			
Grip 1	____________	____________	kg
Grip 2	____________	____________	kg
Pinch 1	____________	____________	kg
Pinch 2	____________	____________	kg
Key 1	____________	____________	kg
Key 2	____________	____________	kg
3JC 1	____________	____________	kg
3JC 2	____________	____________	kg
